# Enhancing sustainability through resource efficiency in beef production systems using a sliding time window-based approach and frame scores

**DOI:** 10.1016/j.heliyon.2023.e17773

**Published:** 2023-07-04

**Authors:** Muhammad Ismail, Tareq Al-Ansari

**Affiliations:** College of Science and Engineering, Hamad Bin Khalifa University, Qatar Foundation, Doha, Qatar

**Keywords:** Livestock, Planning and scheduling, Food security, Food supply chain, Decision support systems

## Abstract

The food needs of the increasing global population, inefficiencies in supply chains, customer expectations and environmental concerns are the challenges to meeting resource-intensive protein needs sustainably. Collectively, this increases the need to enhance sustainability in the beef sector. This study proposes a sliding time-window-based multi-period livestock production model using mixed-integer linear programming (MILP) to simultaneously balance economic and environmental losses. It identifies the optimal finishing time using frame score (FS) and feed conversion ratio (FCR), targeting flexibility by allowing variable growth periods to reduce food/nutritional losses while meeting the variability in demands with minimum inventory levels. Furthermore, sequencing and assigning animals to facilities with optimum separation time is applied to avoid bad handling of animals and ensure quality meat with hygienic standards for longer shelf life. The system boundary of the proposed model includes beef farms and processing facilities. Compared to the recently proposed batch processing models over seven months with a herd size of 1980 animals, the findings reduce the average forage needed by ∼126.90 kips and methane emissions by ∼2560 kg, with a significant benefit in terms of the live animals' weight gain by ∼10,276 lbs.

## 1 Introduction

The increase in global population and the shift towards protein-based foods have increased food security risks [1], leading to intensified agriculture/food production and compounded uncertainties related to waste, water, soil, and air quality. The demand for protein from livestock has increased in emerging economies as countries strive towards an improved quality of life [2]. Additionally, changing rain patterns, droughts, and floods due to climatic changes affect agricultural productivity globally, exacerbating food insecurity. Therefore, to meet the demand for meat and protein, addressing fluctuating costs associated with feeding inputs and livestock management for higher yield requirements in a resource-constrained world is necessary.

A significant portion of nutritional needs essential for human health is directly or indirectly derived from resource-intensive dairy and meat sector products [3]. However, the global livestock sector contributes approximately 14.5% of all anthropogenic GHG emissions, amounting to 7.1 Gt CO_2_-eq/y [4]. Incidentally, beef has the second-highest protein emission intensity per unit after buffalo meat, with an average of 295 kg CO_2_-eq/kg of protein [4]. Multiple factors influence these emissions, including rumination, feed type and quality [5], and the linear flow of water and energy, which contributes to inefficient resource management in production, contributing to these footprints. Furthermore, in addition to feed quality/quantity, the feed type also influences emissions during its digestion in animals.

In cattle farms, generated wastes that harm the environment primarily include emissions, manure, and wastewater [6]. Besides, feed production for livestock also contributes to emissions and eutrophication. Freshwater use in agriculture accounts for roughly 70% of freshwater resources globally [7]. The decreased recharging capacity and excessive drawing of underground water increase deep drilling trends, leading to groundwater depletion and degradation, amplifying negative impacts on water security within the energy-water nexus [8]. The varying approaches for operating cattle farms (barns and pasture-based or mixed) also introduce inefficiencies. In barn-type farms, modification to improved feed helps achieve higher animal growth rates with better control over waste management, helping reduce emissions and wastes significantly at the cost of higher disease risks [6]. However, waste management is almost impossible in pasture-based farms with weak control over feed modification [9], leading to higher variance in growth rates.

**Fig. 1:**
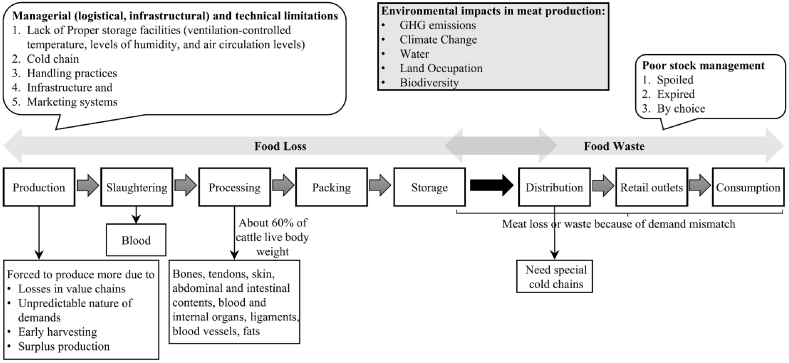
Meat supply chain stages indicating inefficiencies, losses, waste and by-products.

Greenhouse gas (GHG) emissions also arise from food spoilage/waste due to surplus production, leftovers after fulfilling all demands, low quality, spoilage, or poor final product handling [10]. The limitation to avoid spoilage at the farm originates from the storage facilities, as it is essential to maintain ventilation-controlled temperature, humidity, and air circulation levels. The volume of meat waste is ∼4% of the total meat produced; however, its carbon footprint is >20% of the total food waste emissions globally [10]. The negligence in addressing this problem post-slaughtering may increase climate change, wasted operation time and input resources such as water, feed, and energy. In this regard, Fig. 1 compiled from [[11], [12], [13], [14]], illustrates the beef production system and the possible sources of inefficiencies that contribute to food loss and waste at the various stages, which include managerial and technical limitations from slaughtering, processing, packaging, handling practices, cold chain, and marketing system and storage facilities [12]. These inefficiencies, which are manifested in terms of financial losses and environmental impacts, can be controlled and reduced through carefully planned production processes based on market demands.

Within the beef production system, the growth rate of cattle is a complex function dependent on various factors, which directly or indirectly impact both economic and environmental aspects of beef production, making it challenging to accurately determine the expected finishing time of cattle. These factors include Temperature-Humidity Index (THI), genetic composition, weather, feed type and quality, diseases and living space limitations [15]. Furthermore, in post-production, a significant portion of beef is lost during harvesting, processing and packaging due to the aforementioned inefficiencies [16]. Secondly, food losses from non-wealthy farmers are motivated by their needs to meet their living requirements or liquidity, implying an early harvest that leads to a loss in nutritional and economic value [11]. Thirdly, external factors contributing significantly to food losses include the uncertainty, high variance and unpredictable nature of demand patterns in the food sector, leading to surplus production and pushing producers to produce more to satisfy unexpected requirements [11]. Finally, maintaining acceptable quality and hygiene in fresh products, avoiding contamination at any stage and maintaining minimum inventory levels are of the utmost importance, which can be controlled through operational improvements. In this regard, it is imperative to improve productivity and operational efficiency within beef production systems and to do so based on sustainability dimensions.

This study aims to enhance the sustainability performance of beef production and optimise the system by considering the animals' widely varying growth rates due to vast genetic differences. It is envisaged that optimising the system's yield in this manner will reduce the environmental footprint and resource depletion. A further benefit of this enhancement and improved cattle handling includes a higher quality product and longer shelf life [17]. The novelty of the proposed model is to identify the optimal finishing time of the cattle and subsequently schedule the assignment of the animals for processing within the specific time window of finishing period as observed optimum on a case-to-case basis. The proposed model reduces operation times and avoids bad handling of animals and congestion at facilities, eventually leading to improved product quality and longer shelf life. It also supports the lot sizing of various product types for operational excellence and manages the inventory of individual product types to ensure fresh meat supply to the market and reduces food spoilage. After fulfilling all meat requirements, leftover animals that are not feasible to be kept on the farm can be sent to the market for selling as a live product. Considering the above, the objectives of this study are to:

• propose an integrated beef production management system that enhances sustainability through resource efficiency in livestock production by finding the optimal finishing times while applying relevant constraints to help enhance economic gains; and
• support in the selection of a better combination of animals based on optimal target maturity weights to process against high variations in demand and inventory. Also, specifying the processing facility for each cattle avoids infeasible management of animals, load on the plant, and congestion at the subsequent stages.

The remaining sections of this study are organised as follows: section 2 discusses previously published work regarding planning in livestock, used methods, i.e., optimisation tools and solvers used, and research gaps. Section 3 details the methodology, which includes the problem statement and the detailed mathematical formulation to optimise the system, consisting of the objectives and constraints.

## 2 Review of beef production systems: concepts and models

The beef production system, in a broader aspect, is a complex combination of biological processes and operations with inputs, outputs, by-products and waste. The system's primary inputs include livestock feed from crops, land, labour, housing, water, and energy, whereas the outputs include meat, milk, animal skin, and various wastes such as GHG emissions, manure, and wastewater. The complex factors that lead to growth variations in cattle include the daily feed and water intake, which again depends on several factors, including biological variations, forage quality, cattle live body weight, metabolism, THI, activity, and production stage (gestating or lactating). Irrespective of the feed for healthy cows, weight gain positively correlates with feed intake [18]. It shows that extracting fundamental relationships is possible, although tracking these factors' interrelations and complexity is challenging.

Optimising beef production can reduce methane and non-methane emissions. The non-methane emissions of 1 kg of beef are 36 kg CO_2_-eq., approximately 4*x* the impact of 1 kg of chicken and 10–100*x* of plant-origin foods [19]. These non-methane emissions sources include land-use changes, land to grow animal feed, emissions from waste, pasture management (liming, fertilisation and irrigation), and conversion of peat soils to agriculture [20]. In addition to the non-methane emissions above, it is possible to reduce methane emissions by improving Feed Conversion Ratio (FCR) in ruminants [4,21].

It is crucial to ensure nutritional and economic gains against multiple growth-affecting factors and meat loss due to quantity and quality drivers. Meat loss due to quantity includes early slaughtering and spoilage of surplus production after meeting all demands. The meat quality and gains depend on how the animal is treated immediately prior to transportation/slaughtering. If the animal has no stress and is treated well, the expected meat quality is good, whilst the opposite is true [22,23]. Another source affecting quality is contamination during and after slaughtering [24].

Generally, given the livestock sector's inefficiencies, improvements in planning and management are considered the most viable solution to support the harvesting, processing, and packaging of various products more effectively despite limited facility capacity. To enhance resource efficiency and reduce emissions, modifying the operations and processes along the supply chain is essential, and this can be achieved through technology, choosing alternative processes/operations, or optimising current systems. The adopted practices by meat processors with various stages of interdependencies impact the whole chain [25]. The strategies to mitigate such impacts include reduction in food loss and recycling waste [1], improving the FCR [21], and technology adoption against contingencies.

Enhancing the efficiency of the beef production system can be achieved through optimal finishing time, planning, and scheduling sequential/parallel working facilities with inventory management. Adopting relevant standards regarding determining upper bound on capacities of processing and storage facilities reduces beef loss in quantity. From a sustainability perspective, effective planning and scheduling by means of matching the production with market expectations reduce surplus production, ultimately saving resources, reducing emissions, preventing food loss, and enhancing hygiene. Such planning involves monitoring cattle's health and timely finishing by identifying animals' unusual behaviour, eating habits and response to weather changes.

In the design of the operational characteristics of beef production systems, it is important to consider resource limitations, which can arise from three areas: (1) input limitations include allocating pastureland, labour, machine time, water and energy; (2) inside the farms, the herd's genetic differences are a significant factor in continuous variations in animals' feed intake, response to environmental changes, growth rate, dwarfism, and disease tolerance, in addition to living space, slaughtering and processing/packaging facilities capacities (scheduling and max operation times). Moreover, the controlled environment in barn-type farms favours a better growth rate and supports earlier completion of the production cycle; and (3) the general constraints, which include the output storage and inventory capacities, market price, lot sizing of one or several products, and shelf-life considerations. Furthermore, managing the utilisation of resources and optimising productivity amidst externalities such as uncontrolled/unexpected diseases and environmental risks/uncertainties is challenging. Given the various influencing factors, ensuring the animal's optimum size and optimising the finishing time is thus challenging; however, doing so will inevitably draw environmental and economic benefits. For handling such complexities, a variable growth period is required for animals under a specified criterion, which in a broader sense, ensures maximum possible gains from a livestock system by indirectly utilising minimum resources.

Optimum planning and scheduling for beef quality/quantity against demands is necessary to limit nutritional, economic, and environmental losses within beef production systems. Such planning should include the FCR [26] and thus feed optimisation [27], different animal sizes due to genetic variations such as congenital disease dwarfism [28]. Furthermore, slaughtering earlier or later than the optimal time, which usually occurs in batch processing [29] or all-in all-out [24], may incur losses either nutritionally, economically, or environmentally; Furthermore, the congestion and infeasible management at processing facilities ultimately resulting in the mishandling of animals (affecting meat quality), which can be avoided by means of batch lines with a real-time optimisation-based decision system [29]. Simultaneously, these animals' optimal assignment to the facilities for slaughtering and processing helps achieve higher efficiencies and minimum operating times, while the time required (separation time) for protocols ensures hygienic conditions and shelf life [27]. This management may also ease the subsequent stages of animal parts separation, packaging, and stocking in the plant.

Notably, the feed efficiency of the beef production system can be measured using FCR, which is a valuable indicator of economic and environmental performance, defined as [30]:

$$FCR=\frac{Total\;feed\;used}{Unit\;weight\;gain}$$

It can vary from species to species and at different stages of the animal's lifetime, as approximated in several studies [31,32]. Factors that affect feed intake include the environment, husbandry conditions, gene variations and feed type. It also indicates environmental performance as it can specify unwanted outputs and nutrients to the environment [21]. Although helpful, it does not consider the animal's feed content, edible portion (bones to meat ratio), and the final product meat's nutritional quality. These variations are due to the changing growth rates at different life stages, metabolic activity, and genetic variations, which is a challenging factor in quantifying resource inflows against unit gains.

As it is resource-intensive with a considerable footprint, high resource efficiency in beef production is crucial to meet the expected food demands. As farmers have little influence over beef prices, optimal strategies for improving financial results through better herd management, e.g., decisions regarding grazing, feeding level, slaughtering, weighing, growth period to reach full body size, and milk quantity, are essential to maximising gains. However, neglected factors such as genetic differences and uncertainties in climate and diseases can adversely affect overall efficiency, which can be overcome by incorporating these aspects in mathematical models to support optimal decision-making within the livestock sector. In this regard, mixed integer linear programming was proposed using batch lines as an effective livestock planning technique to manage animal growth, indicating an enhancement in the production outputs and a reduction in feed costs [29]. While considering genetic variations amongst animals of the same species, the importance of gender separation, number of animals to be kept in a facility, sequencing and assignment in batch lines with variable growth time in production planning for stable feed and load to the processing plant is considered crucial. Nielsen et al. [33] studied organic beef production systems for optimal economic output and analyzed the results' stability (sensitivity). Mosnier et al. [34] proposed a bio-economic model to simulate and optimise trade-offs amongst various decision alternatives related to forage, crop production alternatives, animal diets, organic fertilisation, and capital. In this case, emissions of feed production (N_2_O, CO_2_) per animal are estimated by considering energy-corrected milk (protein and fats) and per kg of live weight without considering its quality.

To enhance production from each individual animal against all these complex factors, the use of Frame Score (FS) specifying the skeletal size [35], when coupled with other indicators, it can be beneficial. The benefit of using FS is that when the height changes with age, it remains the same for a particular animal throughout that animal's life and is independent of whether the animal is pasture or grain-fed [36]. Determining the FS requires considering both hip height and cattle age. The hip height can be measured directly over the hip bones (hooks) with the animal standing on a level surface. The use of FS also supports the identification of the harvesting time of a particular animal at the farm. FS tables for Angus species are available publicly, which approximate cattle FS based on hip height (inches) for expected target finishing weights (lbs) approved by the Beef Improvement Federation [37].

Considering the inhibiting factors and contingencies related to beef production and extra cattle raising, the need for a system that can tackle such widely varying factors for maximum gains arises. The beef production systems still lack an integrated approach that considers the genetic and other complex factors variations among animals, flexibility in the growth period, lot sizing and operational efficiency of the abattoirs for increased gains. As such, there is an opportunity to address this gap by modelling an integrated, comprehensive system that can provide a rapid and enhanced solution for better management at the farm level. Developing such a decision system at the production stage that integrates increased factors contributes substantially to improving overall benefits against inputs, facilities' utilisation, operation efficiencies; while reducing meat losses and, thus the economic and environmental footprints.

## 3 Methodology

**Fig. 2:**
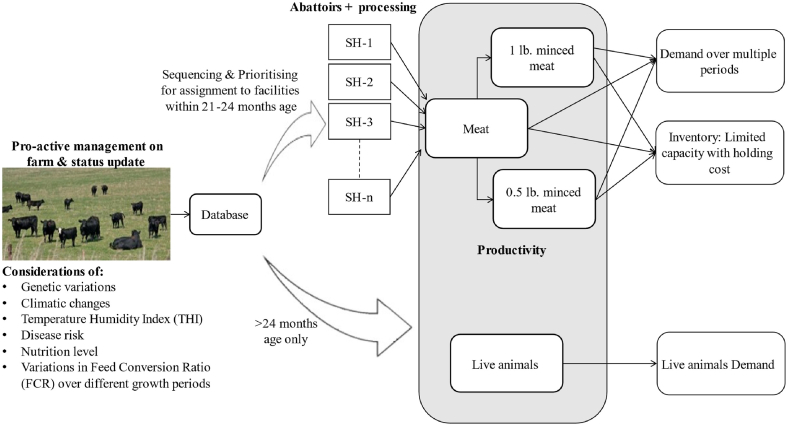
General illustration of beef production system.

The main objective of this study is to develop a relatively straight forward mechanism to determine an animal's near-optimal processing time based on feasible/optimal weight at maturity while ensuring meat flavour and tenderness. To ensure the maximum body size of an animal for maximising the gains, the crucial growth period, referred to as the finishing period, must be kept variable in beef herds due to complex factors. Intensive feedlots feeding or utilising grains is favourable in regard to managing the timely finishing of beef cattle. The target is to get feasible/optimal weight at maturity within a variable-sized window against all the complex linked growth-inhibiting factors. Fig. 2 illustrates the general schematic for a beef production planning system for pasture and grain-fed beef herds. To ensure the maximum body size of an animal for maximising the gains, the crucial growth period must be kept variable in beef herds due to complex factors. The animals can be sold after processing in various meat products or as live animals to fulfil demands. The animals are chosen and prioritised for slaughtering based on FS criterion within 21–24 months of age while closely observing the animal growth. For the FS of an animal, the expected target weight from FS table is used as a determining factor to prioritise and sequence the best-fit animals for slaughtering. The animals that pass fitness are then assigned to multiple facilities for processing to fulfil demands. As the multiple processing units are available inside the farm, transportation of live animals is not considered. The leftover live animals meeting the criteria of unusually high FCR and >24 months of age are sold in the market as per demand at the prevailing price.

The multiple slaughtering facilities are optimised for efficient use to ensure maximum productivity. Introducing a slaughtering cost of the facility usage while matching market needs automatically picks the minimum number and relatively bigger animals for slaughter. Furthermore, to avoid unfavourable incidents from the processing and packaging stages of the supply chain, the model allows for setting a separation time between animals' processing and subsequent steps to improve shelf-life and ensure hygiene. Holding cost applied to the meat inventory stock obtained from the previous period ensures fresh food supply keeping the minimum possible inventory in stock. Summarily, the focus is to specify: 1) an optimum time in a window defined by ready and due time where the model manages operations against demands to achieve the objective; 2) the best suitable set of animals for slaughtering considering customer demands to keep minimum inventory level; and 3) the set of animals are then sequenced for processing at multiple facilities.

### 3.1 Determining finishing period and optimal target weight

A convenient method to assess the skeletal size is by considering the height of a breed at a specific age, which is expressed as Frame Score (FS). Contrary to the models based on the batch principle [24] and all-in all-out [21], this study utilises the actual records of animals' age and hip height to determine the FS, which is then used to determine the corresponding target weight at maturity. It helps approximate the finishing period for an animal by applying a variable-sized sliding window for the varying growth genetic factor. The finishing period is different for every cattle on the farm based on the growth rates in the finishing period.

Two-time instants determine each animal's finishing period window: the ready time and the end/due time. This window size is different from animal to animal due to genetic factor. The proposed model suggests close monitoring for ‘ready time’ just a month prior to the expected target processing time for the possibility of early maturity. In the literature, the target processing time is derived from the population of a species, which is approximately 22 months for Angus beef cattle. For simplicity, this method for monitoring processing time is more practical. Ready time is characterised by growth curves, where differential gains in animal live body weight begin decreasing significantly, whereas its near zero or negative values characterise ‘due time’. Beyond the due time, FCR increases; therefore, keeping animals on the farm is not feasible and must be sold in the market if not processed against demands.

**Fig. 3:**
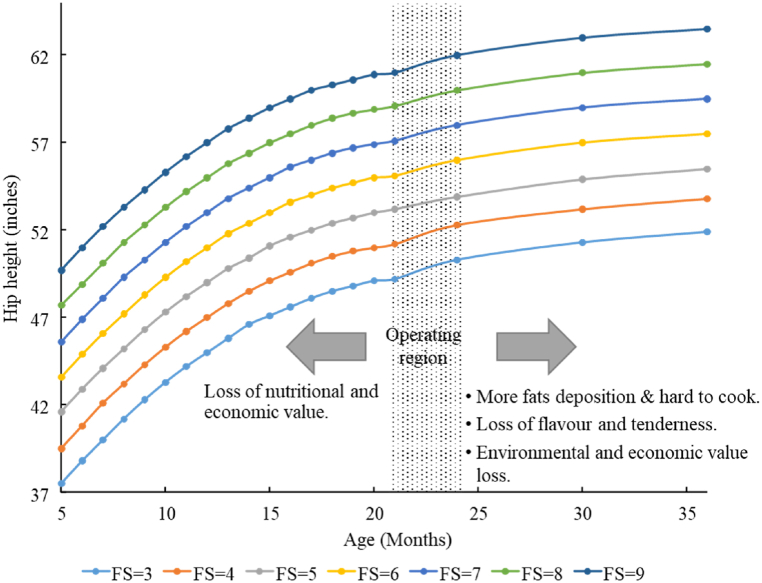
Age vs Hip height for various frame scores and operating region window.

As illustrated in Fig. 3, in the initial months of beef cattle or livestock animals' life, the growth rate (rate of hip height gain and the rate of live body weight gain) is very high with a relatively much lower FCR/relative weight gain than in the later stages of life, and therefore, it is not recommended to slaughter any animal. Over time, when the animal reaches about 80% of its maximum height and weight, a slight decrease in growth rate is observed. The maximum of the FS curve represents the animal's maximum body size with relatively higher fats, which are unsuitable for human health. In beef production, the optimum time of slaughtering varies according to the beef cattle species. This study considers the Angus type, where the age at the peak of the growth curve is about 30 months, although the most beneficial time for the processing lies in between 21 and 24 months when one can expect quality meat with optimum fats, flavour and tenderness [37]. Variations in the mentioned age range depend upon different growth retarding environmental factors during the lifetime. Beyond this period, the hip height begins to reduce slightly, although the live weight still increases, albeit significantly lower. Soon after this condition emerges, the remaining live animals must be sent to the market for selling.

FS can be approximated from “height for age FS tables” or calculated by mathematical formulae. Separate charts for gender exist due to different growth rates. FS being constant throughout a cattle's life, it is an appropriate indicator to inform fitness and harvesting time for processing of a particular animal based on the expected target weight [37]. Cattle with higher FS (6, 7, 8, 9) have a slower maturity rate with a higher gain rate [36]. Following are corresponding equations to determine FS, applicable from 5 to 21 months [37].

$$FS\left(male\right)=0.4878\times \left(H\right)-0.0289\times \left(A\right)+0.00001947\times {\left(A\right)}^{2}+0.0000334\times \left(H\right)\times \left(A\right)-11.548$$

$$FS\left(female\right)=0.4723\times \left(H\right)-0.0239\times \left(A\right)+0.0000146\times {\left(A\right)}^{2}+0.0000759\times \left(H\right)\times \left(A\right)-11.7086$$

where ‘H' is the height in inches and ‘A' is the age in days.

Livestock culling is not optimum when the FCR is below average, and the benefits of differential weight gain are much higher relative to the environmental footprints [38]. However, in practice, due to higher unusual variations in beef demand over time and market conditions, animals are culled earlier than their maturity, which results in a loss of nutritional and economic value. Usually, this occurs when the system is production deficient or risks are much more powerful. On the other hand, with increasing age, the increasing FCR causes the expenses to increase manifold and the cows' differential weight gains become non-significant resulting in higher environmental footprints and economic losses. This situation is faced when there is a surplus raising of cattle without a demand. For this purpose, careful planning based on market history is vital.

**Fig. 4:**
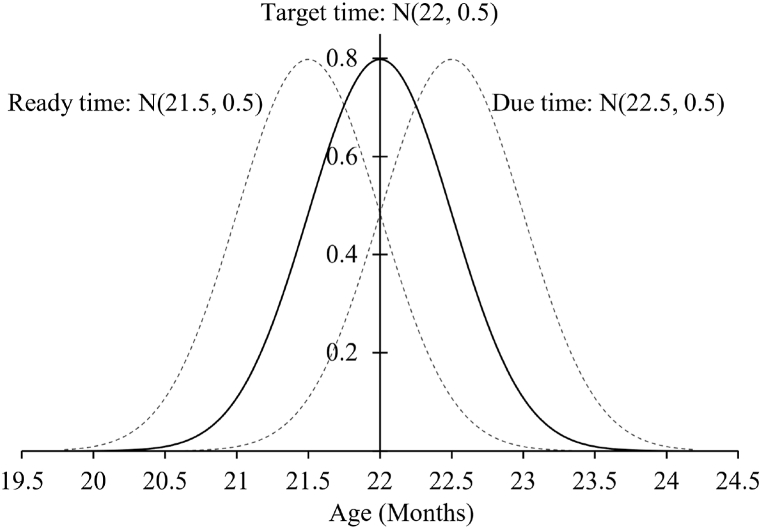
Angus species expected age to reach target live body weight represented as Gaussian (22, 0.5) with ready and due times.

The live body weight of Angus cattle reaches the expected target weight at ∼22 months on average. The age of animals for achieving the target weight at maturity varies with probability because of dependency on genetic variations and environmental factors. For analysis, it is deemed as normal distribution [39], and therefore, the maturity may come earlier or later, in days extending up to a month, as illustrated in Fig. 4. The right and the left doted normal distribution curves represent the possible ready and due time at maturity representing finishing period for analysis purposes [18].

Due to the genetic, breed and age variations, the actual ready time would be a few days earlier than 22 months (mean of maturity age) for every animal, as illustrated in Fig. 4. The ready time is taken as Gaussian N (21.5,0.5) to consider the risks and genetic factors for analysis. In practice, when an animal reaches its expected ready time (∼21 months), it should be monitored closely for any deviations in differential weight gain and the feed quantity it takes to evaluate the FCR along with the growth charts (for health/optimum growth). Significant deviations from growth charts or a relatively higher differential increase in FCR indicates ready time. After this time, the animal must be sequenced and assigned to a slaughterhouse. The expected target processing time of 22 months varies and needs adjustments from species to species. Generally, the actual ready time and due time must lie within 21–24 months for Angus beef cattle. However, the mean and variance may vary from region to region due to the current farm management practices.

Similarly, the due time is also considered Gaussian but at least more than the ready time. Also, for analysis, market demand can be considered Gaussian with fixed mean and changing variance [[40], [41], [42]], but in practice, it can be determined using forecasting techniques. In Excel, the random number generation function under “Data analysis” was used to generate the Gaussian random data and afterwards rounded the values to the nearest multiples of tens (per pack size).

### 3.2 Problem statement

Given a set of animals having wide genetic variations and ages *A* = *{1, …,n}*, multiple processing facilities *S* = *{1, …,m}* with specific operation times for the production of various product types *G* = *{1, …,q}* supply and storage over multiple periods *T* = *{1, …,p}*. The animals are either processed and sold as meat or meat products and live animals. The focus is determining the best balance for sustainability by maximising gains against genetic variations, limited facilities and storage capacities irrespective of the geographical region, size and type of farms. The assignment of animals to limited processing facilities is scheduled based on the overall gains' maximisation and associated costs/penalties reduction. For details of all the mathematical notations, parameters and decision variables, see Table 1.

**Table 1:** Mathematical notations, parameters and variables used in this paper.

Sets:	
A:	Set of all the animals available at farm, *A* = *{1, …, n}*.
S:	Set of slaughter houses, *S* = *{1, …, m}*.
T	Set of periods with time step of 1 month, *T* = *{1, …,p}*.
G	Set of product types produced, *G* = *{1, …,q}*.
Parameters:	
${TWA}_{a,t}$	Array of live target weight of animal a (lbs) with expected fitness for period t. a∈A,t∈T.
M	A constant big number taken 3000 for this analysis.
LOC	Loss of opportunity cost.
C	Constant, representing the mean time required for cleaning facilities.
ST	Average Separation time between the slaughtering of animals.
$R_{a,t}$	Expected remaining days to slaughter animal a in period t, where a∈A,t∈T.
${\mathrm{Pr}}_{g}$	Price for meat product types g, where g∈G.
${\mathrm{Pr}}^{live}$	Price/lb for remaining live animals after fulfilling meat products demand.
${Prt}^{lbs}$	Processing time required for one lb of meat processing.
${Prop}_{g}$	Proportion of meat required to produce each type of specific output product.
$D_{a,t}$	Due time of animal a in period t, where a∈A,t∈T.
${Req}_{g,t}$	Demand for meat products of type g, expected for period t, where g∈G,t∈T.
${INV_{cap}}_{g}^{meat}$	Array containing maximum units of meat inventory capacity of product type g based on facility conditions.
${HC}_{g}^{meat}$	Holding cost per unit of g type of product inventory.
$P^{+}$:	Penalty of late slaughtering in $ for environmental and economic losses.
$P^{-}$	Penalty of early slaughtering in $ due to nutritional and economic losses.
$c_{a}^{s}$	Cost of slaughtering per animal a, where a∈A.
Variables:	
$x_{a,t}$	Actual assigned slaughtering time of animal *a* in period *t*, where a∈A,t∈T.
$z_{a,b,t}$	Whether animals *a* & *b* slaughtered in the same slaughterhouse in period *t*, a,b∈A,a≠b.
$y_{a,b,t}$	Whether animal *a* slaughtered earlier than animal *b* in period t, a,b∈A,t∈T,a≠b.
$\gamma_{a,s,t}$	Whether animal *a* is assigned to slaughterhouse s in period *t*, a∈A, s∈S,t∈T.
$h_{g,t}^{meat}$	Inventory of meat product type *g* at the start of period *t*, where g∈G,t∈T
${Req_{serv}}_{g,t}\text{:}$	Requirements served for different meat products of type *g* in period *t*, where g∈G,t∈T.
${Rem_{live}}_{a,t}^{sold}$	Leftover older live animals *a* sold in period *t* after meeting meat product.
${Rem_{live}}_{a,t}^{inv}$	Remaining live animals' inventory at the end of the time horizon.
$\alpha_{a,t}$	How much earlier is an animal *a* slaughtered in a period *t* from ready time, a∈A,t∈T.
$\beta_{a,t}$:	How much later an animal *a* slaughtered in a period *t* after due time, a∈A,t∈T.
$\alpha_{a,t}=\max\left(0,R_{a,t}-x_{a,t}\right)$	Early slaughtering of animal a in period t, a∈A,t∈T.
$\beta_{a,t}=\max\left(0,x_{a,t}-D_{a,t}\right)$	Late slaughtering of animal a in period t, a∈A,t∈T.
$B{TW}_{i}=\max\left(0,x_{a}-R_{a,t},D_{a,t}-x_{a,t}\right)$	Best interval of slaughtering (as symmetric uniform distribution) without any penalty.

Keeping in view the growth curve and the genetic variations, every animal has its own ready/finishing time $R_{a,t}$ and due time $D_{a,t}$ which usually deviates from the target slaughtering time. Both follow the Gaussian normal distribution and ‘Ready-time’ must always be less than ‘Due-time’. The animals not processed to fulfil meat demands and exceeding 24 months of age with an unusual increase in FCR which means more contribution in costs/penalties indicate environmental and economic performance degradation [21], and it is no more feasible to keep those animals on the farm. The deviations penalties for processing while meeting demands are based on either an early assignment of animal *a,* given by $\alpha_{a,t}=\max\left(0,R_{a,t}-x_{a,t}\right)$ or late assignment of animal *a* given by $\beta_{a,t}=\max\left(0,x_{a,t}-D_{a,t}\right)$, where $x_{a,t}$ is the optimised assigned time for an animal. In between $R_{a,t}$ and $D_{a,t}$ is the best time for processing. While multiple animals fit the above criteria and lie in the best time window, they are sequenced for processing to avoid congestion, overloading facilities, and mishandling of animals.

While fulfilling demands, the multiple products' inventory information is maintained separately for each product. The assignment of processing time in the same facility is separated for each animal based on the linear function of its live body weight and a setup time to ensure hygienic conditions and shelf-life improvement. Contrary to batch processing, if the meat products are tagged for identification, the losses will be limited as it can help reduce both food and economic losses when recalling is needed. Only a particular animal's meat will be recalled instead of whole batches.

### 3.3 Formulation

The objective function used in the mixed-integer linear programming (MILP) problem finds a balance between the net economic, nutritional, and environmental gains while fulfilling protein needs by improving the production system's outputs. Equation (1) illustrates the net revenue for the model, where the target is to maximise *Q*.

(1) $$\begin{array}{c} Obj\;Q=\sum_{t=1}^{p}\sum_{g=1}^{q}\left({Req_{serv}}_{g,t}\times {\mathrm{Pr}}_{g}\right)+\sum_{t=1}^{p}\sum_{a=1}^{n}{(Rem_{live}}_{a,t}^{sold}\times {\mathrm{Pr}}_{live})-\\ \sum_{t=1}^{p}\sum_{a=1}^{n}\left(\alpha_{a,t}\times P^{-}+\beta_{i,t}\times P^{+}\right)-\sum_{t=1}^{p}\sum_{g=1}^{q}\left(h_{g,t}^{meat}\times {HC}_{g}^{meat}\right)-\\ \sum_{t=1}^{p}\sum_{s=1}^{m}\sum_{a=1}^{n}\left(\gamma_{a,s,t}\times c_{a}^{s}\right)-LOC\times \sum_{t=1}^{p}\sum_{g=1}^{q}\left({Req}_{g,t}-{Req_{serv}}_{g,t}\right)\;\# \end{array}$$

This objective function has six parts: the first two are the benefits and the rest are the costs incurred. The first part is related to the revenue contribution from selling meat with different lot sizing of minced meat packs (one lb and half lb) and whole meat packs (one lb); the second part represents the revenue from selling remaining live animals in the market. These animals have gained enough size (meat quantity) but contribute relatively more to the environmental footprints depicted by FCR at earlier stages of life and, therefore not feasible to be kept on the farm and not in range of the optimal time window; the third part is related to the costs of earliness and delay in slaughtering. The early slaughtering depicting less live animal body weight with relatively lower FCR causes loss economically and nutritionally. Late slaughtering causes economic and environmental losses as the growth curve becomes flat with an unusual increase in FCR. The fourth part represents inventory holding cost. The fifth part represents the processing facility expenses, and the last one represents the loss of opportunity cost to control the fulfilment of meat requirements.

The penalties/costs can be expressed as either real or complex components spanning sustainability dimensions. The fourth part represents the meat inventory holding cost. The fifth part represents total slaughtering, processing, and packing cost, prioritising the fat animals to be processed first. Finally, the sixth part ensures to save in the loss of opportunity cost. Following are the constraints applied.

(2) $$\begin{array}{c} \left(x_{b,t}-x_{a,t}\right)\geq \left({Prt}^{lbs}\times {TWA}_{a,t}+C\right)\times z_{a,b,t}-M\times y_{b,a,t}\forall a,b\in A,t\in T,a\neq b \end{array}$$

Inequality 2 ensures the separation time between any two animals *a* and *b*; if slaughtered and processed in the same facility for any period *t*, the separation time is represented as a function of processing and packing times dependent on an animal's live body weight and cleaning of facilities represented by $\left({Prt}^{lbs}\times {TWA}_{a,t}+C\right)$. It plays a role in ensuring overall hygienic conditions and shelf-life improvement. Here, optimal scheduling also increases the overall throughput, reducing lead times.

(3) $$\begin{array}{c} z_{a,b,t}=z_{b,a,t}\forall a,b\in A,t\in T,a\neq b \end{array}$$

Equation (3) represents that if animal *a* is slaughtered in the same slaughterhouse as animal *b*, then the same is true the other way for all periods. This forces *z* to be symmetric.

(4) $$\begin{array}{c} y_{a,b,t}+y_{b,a,t}\leq 1\forall a,b\in A,t\in T,a\neq b\;\# \end{array}$$

Inequality 4 ensures that if animal *a* is assigned before *b*, then *b* before *a* is impossible in any period *t*.

(5) $$\begin{array}{c} z_{a,b,t}\geq \gamma_{a,s,t}+\gamma_{b,s,t}-1\forall a,b\in A,s\in S,t\in T,a\neq b \end{array}$$

Inequality 5 links the binary decision variables $z_{a,b,t}$ and $\gamma_{a,s,t}$, where $\gamma_{a,s,t}$ represents animal ‘*a'* slaughtered at facility ‘*s'* in period ‘*t'*.

(6) $$\begin{array}{c} \sum_{s=1}^{m}\gamma_{a,s,t}\leq 1\forall a\in A,s\in S,t\in T \end{array}$$

Inequality 6 ensures avoiding the repetition of slaughtering an animal for all the periods and slaughterhouses.

(7) $$\begin{array}{c} {Req_{serv}}_{g,t}\leq {Prop}_{g}\times {Req}_{g,t}\forall g\in G,t\in T \end{array}$$

Inequality 7 represents that demand served must always be less than or equal to the actual demands. It also incorporates the proportion of meat needed to make one unit of the required product.

(8) $$\begin{array}{c} \sum_{g=1}^{q}h_{g,t-2}^{meat}+0.5\times \sum_{a=1}^{n}\left(\begin{array}{c} \left({TWA}_{a,t-1}\times \sum_{s=1}^{m}\gamma_{a,s,t-1}\right)+\\ \left({TWA}_{a,t}\times \sum_{s=1}^{m}\gamma_{a,s,t}\right)+\\ \left({TWA}_{a,t+1}\times \sum_{s=1}^{m}\gamma_{a,s,t+1}\right) \end{array}\right)=\\ \sum_{g=1}^{q}\left({Req_{serv}}_{g,t}\right)+\sum_{g=1}^{q}h_{g,t}^{meat}\forall a\in A,s\in S,t\in T,g\in G \end{array}$$

Equation (8) represents the sliding time window of three months (21–24 months age), improving productivity and flexibility against genetic variations and other risks. It also provides the flexibility to serve high variations in demand. Multiplication factor 0.5 represents the 50% edible portion of the live body weight, as the beef cattle's inedible part comprises approximately 50–60% of the animal's live body weight [14]. It also keeps track of various meat products' inventory after fulfilling productivity requirements according to the demands.

The animals whose ‘ready time’ is delayed due to uncertainties and lower FCR must not be slaughtered, which implies profitable growth. These animals must be adjusted for processing in the subsequent periods (22–24 months). However, some animals have gained their maximum weight in earlier periods than expected and can fulfil the earlier period's demands. Therefore, the ready times in the adjacent periods may have some overlapping duration with the immediately previous period.

(9) $$\begin{array}{c} h_{g,t}^{meat}\leq {INV_{cap}}_{g}^{meat}\forall g\in G,t\in T \end{array}$$

Inequality 9 puts a limitation on the inventory of various products produced. This limitation comes from the storage facilities management due to its capacity or fresh food needs when following the standard storage requirements. In addition, these facilities need to maintain a specific temperature and humidity to avoid food loss or spoilage.

(10) $$\begin{array}{c} {TWA}_{a,t}*\left(1-\sum_{s=1}^{m}\gamma_{a,s,t-2}\right)={Rem_{live}}_{a,t}^{sold}\forall a\in A,t\in T,t>2 \end{array}$$

Equation (10) determines the remaining live animals sold after 24 months of age, which lies in the growth curve's nearly flat region with increased FCR.

(11) $$\begin{array}{c} {TWA}_{a,t}*\left(1-\sum_{s=1}^{m}\gamma_{a,s,t}\right)={Rem_{live}}_{a,t}^{inv}\forall a\in A,t\in T,\left(p-1\right)\leq t\leq p \end{array}$$

Equation (11) determines the remaining live animals' closing inventory after the end of the time horizon, which corresponds to only the last two periods (period = month) of the time horizon, considering the time window of productivity.

(12) $$\begin{array}{c} \sum_{t=1}^{p}\sum_{a=1}^{n}\left(\begin{array}{c} {Rem_{live}}_{a,t}^{sold}+\\ {Rem_{live}}_{a,t}^{inv}+\\ \sum_{s=1}^{m}\gamma_{a,s,t}*{TWA}_{a,t} \end{array}\right)=\sum_{t=1}^{p}\sum_{a=1}^{n}{TWA}_{a,t}\\ \forall a\in A,s\in S,t\in T,g\in G\;\# \end{array}$$

Equation (12) ensures that the total number of animals on the farm equals all the animals slaughtered plus the remaining live animals sold and the closing inventory of live animals. The parameters may vary and need adjustments according to the actual data and observations in practice. A farm of 1980 animals with different FS and ages is considered for analysis and demonstration purposes. It is assumed that no animal is brought and included in the herds from outside and all the processing and storage facilities exist close to the farm. The hip height is measured whenever an animal reaches the age of five months on the farm. The expected target weight for that animal is computed using the FS formula for both heifers and steers for future planning and scheduling purposes. The expected finish time for every animal is taken as 22 months, and it must be slaughtered before the expected due time of 24 months. After 24 months, the growth curve is relatively flat, and it is not beneficial to keep these animals on the farm for a more extended period. Due to model complexity, the transportation of live animals for processing and moving meat products to inventory is not considered and assumed that all the facilities exist before the farm gate.

## 4 Results and discussion

An integrated approach within the beef production system is presented that considers genetic variations among animals by providing flexibility in the growth period, considering lot sizing and operational efficiency of abattoirs. The approach is applicable to any species of beef cattle farms irrespective of size and geographic location by setting a few parameters specific to the region or geographic area under consideration. To demonstrate the proposed model's performance, a herd size of 1980 Angus species cattle (assuming90animalsreachmaturityeverymonth×22months), having steers and heifers of different ages and FS, is considered. The applicability to other species needs relevant FS tables. However, the resulting gains or benefits may vary according to regional parameters. The results indicate that the proposed time-window-based model performs better than the recently proposed batch process models (90 animals/batch/month). Furthermore, this approach saves resources by considering an individual animal's target weights corresponding to FS, FCR and fitness for slaughtering.

The model's revenue sources (the outputs) consist of three different kinds of products and the selling of those live animals that are no more beneficial to keep on the farm. The products include 1 lb and 0.5 lb minced meat packs and 1 lb meat (without bones). It needs 1, 0.5 and 1 lb of meat, respectively, to make a unit of each product. About 50% of live animal weight consists of tendons, bones, ligaments, and vessels [14]. This study assumes 50% of the animal's body weight as inedible parts. Therefore, productivity corresponds to the good edible portion of 50% of the animal's live weight. For analysis, each day's operation time is restricted to eight hours to serve the population's maximum possible needs with limited plant capacities. For all the products, the initial inventory is taken as zero. Moreover, the inventory of all three products is maintained and optimised separately for every period.

The model was demonstrated based on a seven-month time horizon with a time step of one month for optimisation purposes. The time step of one month is applicable, especially near maturity, as the differential gain in an animal's live body weight per day is insignificant. After 21 months of age, more frequent weight monitoring and status updates are essential to match the target weight with the real observed one updated in the database to get the most optimised results. The higher update frequency can further help in saving resources. It took about 6 min and 50 s without any heuristics to reach the optimal solution using IBM ILOG CPLEX Optimisation studio version 12.7.1.0 with intel core i7-8700 CPU machine, 16 GB RAM with default settings. Going into details, it consists of 25,166 nodes with 113,400 binary variables, 1305 integer variables, and 8820 float variables with 811,203 non-zero coefficients, as illustrated in Table 2. No conflict amongst the 287,956 constraints is observed.

**Table 2:** CPLEX statistics of the proposed model.

Statistic	Value
Constraints	287,956
Variables	123,525
Binary	113,400
Integer	1305
Other	8820
Non-zero coefficients	811,203
Nodes	25,166
Iterations	845,896

**Fig. 5:**
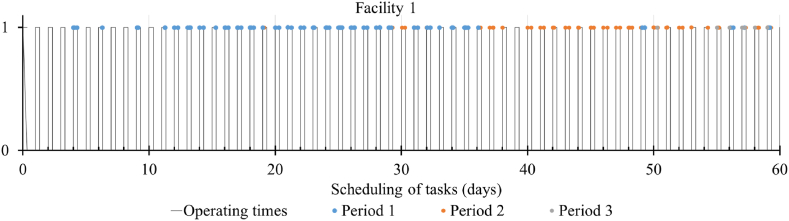
Assignment of animals to one of the facilities for processing where ‘1’ on the vertical axis indicates the availability of the facility during the regular eight working hours.

Fig. 5 illustrates the optimal assignment time of animals to one of the processing facilities for multi-period scheduling within the 8 h/day operating time. The horizontal axis depicts the scheduling of tasks in days, and the vertical axis depicts the availability of the processing facility. The plotted data includes only animals expected to reach their target weight at maturity in the coming 60 days period. A step function ‘stepwise’ in CPLEX was used for task assignment to facilities during regular operation times (8 h/day in this model) or overtime afterwards. The regular operating time and overtime expense can be incorporated by multiplying with the step function. Here, processing an animal into meat involves various sequential stages. The stage which takes the maximum time can be considered the separation time, which is taken as 1 h. Moreover, based on the factors described earlier, the animal's actual assignment time for processing deviates from the expected target time of 22 months by ±30 days, reflected in Fig. 5.

In order to estimate the resource savings/improvements compared to batch processing, the solution obtained from the CPLEX solver was used to determine resources saved by finding the deviations from the optimum slaughtering time compared to the recently proposed batch processing models (one batch/month). Research indicates that mean GHG emissions intensity estimates vary in beef production systems. An estimate of beef emissions taken as a reference for environmental performance analysis is ∼150 kg CH_4_/year/cow, of which 130 kg was due to enteric fermentation and 20 kg from manure and another 58 kg from the replacement heifer [43]. According to this reference, analysis indicates that the system indirectly saves significantly valuable resources compared to the batch processing of livestock. For 1980 animals farm, it saves GHG emissions by approximately ∼2.5 tons of CH_4_ emissions per year, with a gain of ∼9520lbs of extra live animal body weight per year. Moreover, the model also has additional economic and environmental benefits in forage saving amounting to ∼120 tons annually by ensuring optimal animal body size against multiple factors.

While analysing, the feed intake or forage consumed per day is considered a linear function of the animal's live body weight. The cow weighing 1,200lbs needs 24lbs of forage/head/day, having 88% of dry matter [44]. According to this reference, ∼126.90 k-lbs of forage is saved with ∼2560 kg GHG emissions approximately over the seven periods horizon. Moreover, the compromised weight due to early slaughtering in batch processing was estimated to be ∼10,276 lbs in the seven periods horizon. It illustrates the need to raise more animals to meet market requirements, leading to the increased operating time of the facilities and the loss of nutritional, economic, and environmental value.

**Fig. 6:**
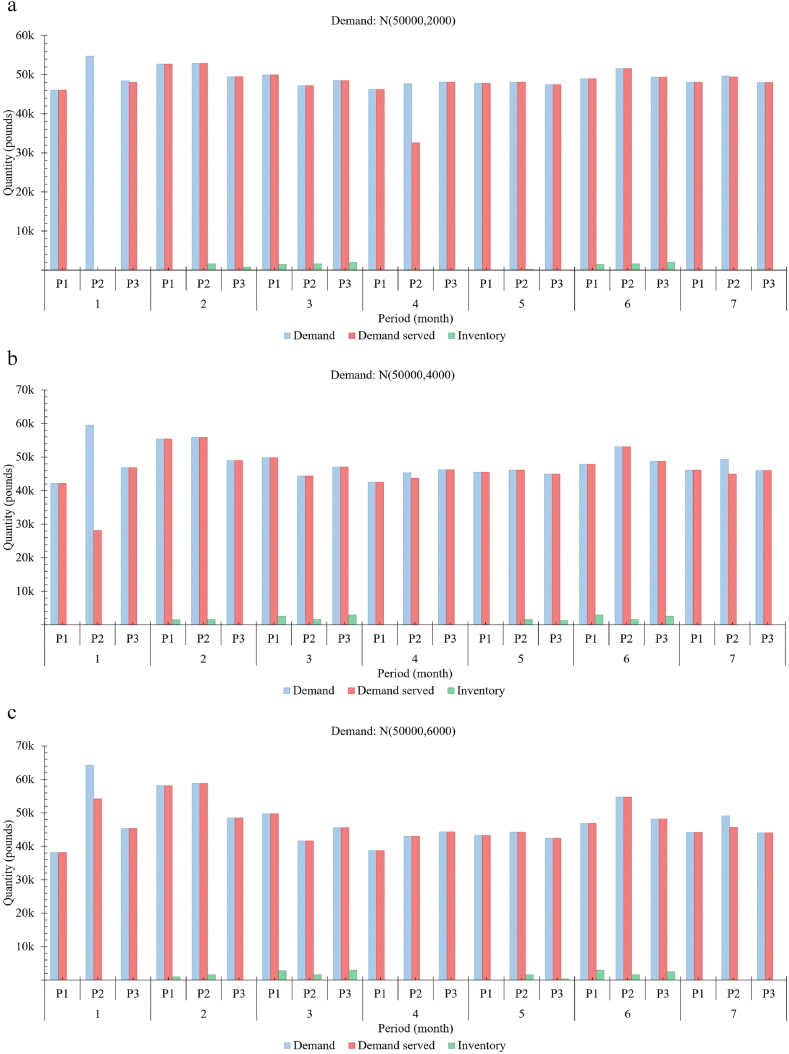

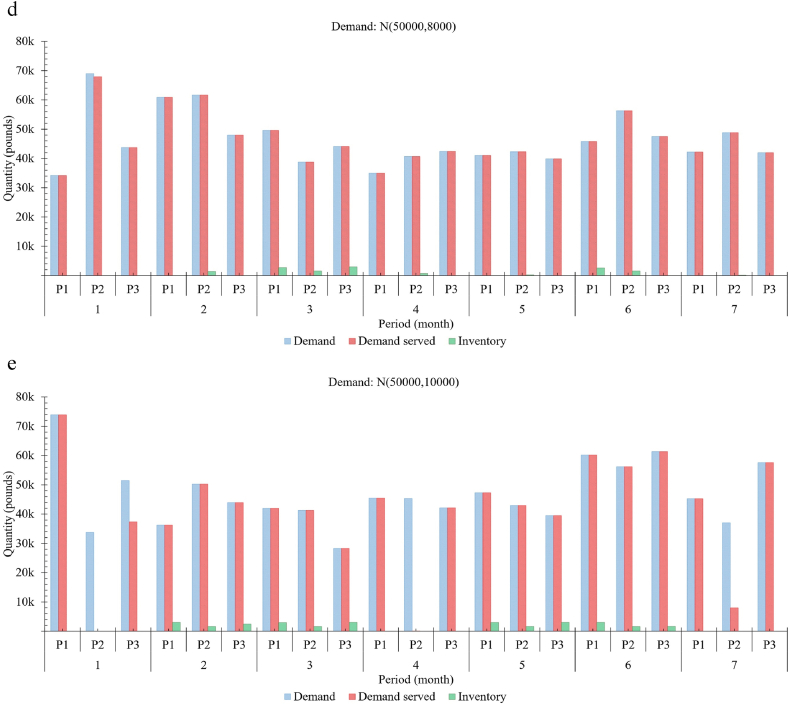
Requirements and fulfilment rate with inventory management. (a–e) Illustrates the response of the model for varying requirements N (50,000,2000), N (50,000, 4000), N (50,000, 6000), N (50,000, 8000) and N (50,000, 10,000) respectively.

Fig. 6(a–e) collectively illustrates the actual demand and demand served with inventory levels for three different meat products, which includes P1: 0.5 l b minced meat, P2: 1.0 l b minced meat and P3: 1.0 l b meat over seven months period. Also, it depicts the proposed model's performance that provides insights to support decision-making while planning farm expansion in terms of herd size and processing facilities. The ‘demand’ here means collective orders received at the start of each month for each product, whereas ‘demand served’ refers to the units provided against those orders. For analysis, the demand for each meat product is generated using Gaussian, having a fixed mean of 50 k pounds of meat with different variances. Here, the mean of 50 k pounds is the approximate capacity of the beef farm. Fig. 6(a–e) illustrates the response of the model for the generated demands N (50,000,2000), N (50,000, 4000), N (50,000, 6000), N (50,000, 8000) and N (50,000, 10,000), respectively. In practice, data-driven demand prediction/forecasting can be used. The demand served and inventory level for each product are recorded to indicate flexibility in the fill rate against each generated demand. This figure also proves that the model provides relatively more resilience to the widely varying nature of demands for food, especially in beef supply chains.

Fig. 6 also depicts the performance and flexibility of three different products requirements fulfilment against highly varying demands. Moreover, this model can help extract valuable information that provides insights regarding optimal farm sizing for better overall performance by considering the adequacy of slaughtering/meat processing facilities and adjusting the farm size to meet the near-future regional beef requirements. Market prices, loss of opportunity $\left({Req}_{g,t}-{Req_{serv}}_{g,t}\right)$ cost and early/late slaughtering costs influence such decisions. For instance,

• Observing both higher loss of opportunity for meat products and increasing number of animals exceeding 24 months of age, and
  - the processing facilities having no slot in the month's operating times infers either to increase the operating hours (over_time:extra_cost) or the number of facilities (CapEx + OpEx), a trade-off.
  - the processing facilities have empty slots in the month's operating times infers either to increase the marketing expense or reduce the prices to adjust supply, a trade-off.
• Frequent loss of opportunity with no animals reaching 24 months infers an increase in farm size.

In practice, the feasibility of packaging some specific quantities is not recommended due to economic reasons as it incurs a relatively higher cost due to packaging material type or noneconomic packaging quantity. For analysis purposes and to observe the effects of such products, a relatively higher production cost is set for the product (P2), which is reflected significantly in Fig. 6. The least priority is given to such products and not even fulfilled in some cases, e.g., graph 1 (periods 1 and 4), graph 2 (period 1), and graph 5 (periods 1, 4 and 7). The graphs collectively illustrate the capability of the model to avoid the loss of opportunity (difference between demand and demand served) significantly. Moreover, these graphs indicate the system's flexibility to fulfil the widely varying demand patterns for food products with minimum inventory levels and avoid surplus production. Inherently, the model's capability to reduce food loss and waste risks while fulfilling beef requirements further improves environmental footprints and resource utilisation.

## 5 Conclusion

The methodology provides an integrated, practical and effective approach that ensures near-optimal finishing time of Angus cattle to maximise overall economic gains. The sliding time window approach accommodates variable growth periods for cattle against genetic variations, feed intake and environmental factors affecting growth rate. The model supports managerial decisions regarding selecting a better combination of animals to process, facilities usage, planning and operational times while ensuring a high fulfilment rate against widely varying demands. Based on FS, the proposed model is independent of farm type and geographical location. The FS-based expected target weight coupled with FCR proved helpful in determining the optimum culling time. The analysis revealed higher gains by identifying the proper time for sequencing and assignment to abattoirs subject to facilities' limitations and associated costs while fulfilling demands ensuring improved nutritional and economic value. Monitoring for FS-based expected target weight and insights from the model favour overall cost reduction concerning various aspects of beef production under the farmer's control. Generalising the model to accommodate different species compromises accuracy to a greater extent due to the variable growth period and operating window size and is not recommended. Furthermore, for the validity of this model, all facilities must exist within the farm boundary, including animal housing, barns or pasture facilities, slaughtering, packing, and inventory/storage.

The model can maintain minimum inventory levels, avoiding food storage costs and food loss and significantly improving the overall sustainability performance. Environmental benefits in land-use change, eutrophication and biodiversity are, though not considered explicitly, inherently benefited by optimising for improved output-to-input ratio through finding the near-optimal time of slaughtering for an individual animal. Although all such factors can be approximated to determine the total environmental benefits, this study only uses methane-related calculations for illustration purposes. Apart from all these benefits, it also saves all the related input resources such as energy, water, feed, labour etc. Also, it promotes the efforts for expanding research and development in the direction of highly reduced emissions with strict constraints in large-scale commercial beef cattle farming settings. In addition to the above, the parameter representing limited capacities of meat products inventory in the storage facilities can be specified depending on the actual fresh food supply and demand scenarios to avoid spoilage or food loss. Multiple possibilities for the continuity of this work exist, although it increases system complexity and becomes challenging to solve. For instance, one can integrate inventory management with capacitated routing for the distribution of the end products as per market requirements.

## Author contribution statement

Muhammad Ismail: Conceived and designed the experiments; Performed the experiments; Analyzed and interpreted the data; Contributed reagents, materials, analysis tools or data; Wrote the paper. Tareq Al-Ansari: Conceived and designed the experiments; Analyzed and interpreted the data; Contributed reagents, materials, analysis tools or data. Data availability statement: Data will be made available on request. Declaration of interest's statement: The authors declare that they have no known competing financial interests or personal relationships that could have appeared to influence the work reported in this paper.

## Declaration of competing interest

The authors declare that they have no known competing financial interests or personal relationships that could have appeared to influence the work reported in this paper.
